# Immune activation and microbial translocation in liver disease progression in HIV/hepatitis co-infected patients: results from the Icona Foundation study

**DOI:** 10.1186/1471-2334-14-79

**Published:** 2014-02-12

**Authors:** Giulia Marchetti, Alessandro Cozzi-Lepri, Camilla Tincati, Andrea Calcagno, Francesca Ceccherini-Silberstein, Andrea De Luca, Andrea Antinori, Antonella Castagna, Massimo Puoti, Antonella d’Arminio Monforte

**Affiliations:** 1 Department of Health Sciences– Clinic of Infectious Diseases – “San Paolo” Hospital, University of Milan, via A. di Rudinì, 8-20142 Milan, Italy; 2 Department of Infection & Population Health Division of Population Health Hampstead Campus, University College London, London, UK; 3 Unit of Infectious Diseases, Department of Medical Sciences, University of Torino, Torino, Italy; 4 Department of Experimental Medicine and Surgery, University of Rome Tor Vergata, Rome, Italy; 5 UO Malattie Infettive, Azienda Ospedaliera Universitari Senese Ospedale Santa Maria alle Scotte, Siena, Italy; 6 Divisione Malattie Infettive I.N.M.I. “L. Spallanzani” I.R.C.C.S., Roma, Italy; 7 Department of Infectious and Tropical Diseases San Raffaele Scientific Institute, Milan, Italy; 8 Infectious Diseases Department, AO Ospedale Niguarda Cà Granda, Milano, Italy

**Keywords:** Microbial translocation, HIV/hepatitis co-infection, sCD14, Fib-4

## Abstract

**Background:**

We evaluated whether immune activation (IA) and microbial translocation (MT) might play a role in accelerating liver disease progression in HIV-HBV/HCV co-infected patients.

**Methods:**

ART-naïve HIV/viral hepatitis co-infected patients from Icona with a CD4 cell count >200/μl and with a known date of prior HIV neg/pos tests and ≥1 plasma sample stored were included in the study. Plasma MT (LPS, sCD14) and IA (IL-6,TNFα) were measured using ELISA while activated CD8 + CD38 + HLA-DR + were measured by flow cytometry, with one measurement being performed for all patients and two measurements for a smaller group of subjects. The association between these biomarkers and the time to i) a single ALT >200 IU/l and ii) a Fib-4 >1.45 was also investigated. A standard survival analysis with robust standard errors was used for all evaluations. Follow-up was censored at patients’ last clinical follow-up.

**Results:**

We studied 127 HIV-infected hepatitis viruses co-infected patients (118 HCV, 9 HBV). Overall median (IQR) CD4, VL, age were 596/μl (208–1303), 3.8 log_10_cp/mL (3–4.3), 34 years (22–56). While heightened TNF-α was associated with a 13-fold increased risk of Fib-4 > 1.45 (RH 13.05, 95% CI 2.43-70; p = 0.003), markers of MT did not show an association with liver illness. Interestingly, higher sCD14 was associated with a decreased risk of Fib-4 > 1.45, independently of other biomarkers considered (RH 0.20, 95% CI 0.04-0,9; p = 0.04).

**Conclusions:**

In HIV/hepatitis virus co-infected ART-naive patients, higher TNF-α plasma levels were associated with a 13-fold increase in the risk of progression to a Fib-4 >1.45, suggesting that the pro-inflammatory status in HIV infection might hasten the course of HCV. In view of the fact that sCD14 may hinder the interaction between LPS and the phagocyte membrane CD14, we herewith propose a model which aims to demonstrate that high sCD14 levels might contribute to shelter liver function through the down-regulation of the inflammatory cascade.

## Background

HBV/HCV co-infection with HIV is known to accelerate liver disease progression, however, the precise mechanisms by which this occurs have yet to be fully elucidated.

Amongst numerous causes, microbial translocation (MT) from the gut has been shown to substantially contribute to liver disease in several clinical settings such as alcoholic liver disease, and other enteric processes [1-4].

In chronic HBV and HCV infection, sCD14 levels differentiate HBV/HCV-infected subjects with severe liver fibrosis from those with minimal fibrosis, and are associated with the risk of failure to respond to anti-HCV treatment with pegylated-interferon-α/ribavirin, suggesting that the host response to MT might predict the outcome of the disease [5].

In HIV infection, MT has been linked to immune activation (IA) [6] and correlates with the clinical outcome independently of CD4+ counts and HIV RNA levels [7,8]; thus, a common pathogenic role of MT in fuelling viral hepatic illness has been hypothesized. Accordingly, in keeping with these findings, MT has been shown to accelerate liver disease progression in cohorts of HIV-infected and HIV uninfected patients [9-11], and hamper the response to pegylated-interferon-α/ribavirin treatment in HCV/HIV co-infected patients [12]. Most recently, in a retrospective case–control study of HIV + and HIV- Ugandan subjects with detectable liver stiffness/cirrhosis in the absence of HCV infection, Redd *et al.* demonstrated a significant association between monocyte activation and liver disease only in HIV-infected patients, that appeared however unrelated to MT [13].

Given these premises, we investigated whether MT and IA markers might be able to predict the progression of hepatic disease in a cohort of HIV-HBV/HCV co-infected patients.

## Methods

### Study population

This is a nested analysis within a main study in the ICONA Cohort which investigated the association between biomarkers of MT and IA and HIV progression [7]. The study was approved by the Ethical Committee of all the Centers participating to the ICONA Foundation Study (see ackowledgments). All patients signed written consent for use of biological material. Details of the ICONA Foundation Study cohort study and data collection have been previously reported [7].

Briefly, the main study mentioned above was conducted in a subgroup of patients enrolled in the ICONA Foundation Study with a documented date of last HIV-negative and first HIV-positive test, at least one plasma stored while antiretroviral therapy (ART)-naive and a CD4+ cell count greater than 200 cells/l and ALT < 200 IU/L at the date of the stored plasma considered for this analysis. If a patient had more than one sample satisfying these criteria, the less recent sample was used. For some patients 2 samples were used, this less recent sample and another sample collected about 1 year later. In this nested analysis only patients who were tested positive for HCV antibodies or HBV antigens were included.

### Assessment of liver fibrosis

The non-invasive marker of liver fibrosis Fib-4 was calculated as per the recommended formula: age (years) × AST [U/l]/(platelets [10^9^/l] × (ALT [U/l])^1/2^) [14]. For the purpose of our research we decided to use as endpoint a Fib-4 value >1.45, based on literature data showing that a threshold value <1.45 has a negative predictive value for the exclusion of extensive fibrosis (F4-F6 of the Ishak classification) of 90%, and a threshold value >3.25 has a positive predictive value for the diagnosis of extended fibrosis of 65% [14,15]. We defined as events all the patients with a Fib-4 value >1.45 that was confirmed by another value >1.45.

### Assessment of immune activation

Commercially available ELISA kits were used to quantify plasma levels of sCD14, IL-6, and TNF-α (R&D Systems Europe, Abingdon, United Kingdom). The assays were performed in duplicate according to manufacturer’s instructions. In a subset of patients with an available frozen cell sample stored in viable conditions, surface phenotypes were evaluated on thawed PBMCs by flow cytometry (FACS Sort Becton-Dickinson, San Josè, California, USA) using directly labeled antibodies (fluorescein isothiocyanate [FITC], phycoerytrin [PE], and Peridinin-chlorophyll-protein complex cyanin 5.5 [PerCP Cy5.5]). We determined CD8+ T-lymphocyte activation by measuring the expression of HLA-DR and CD38. The following combination was used: CD8/CD38/HLA-DR (CD38-PE, HLA-DR-FITC CD8-PerCP Cy5.5, Becton Dickinson, San Josè, CA, USA). All biomarkers were measured in a central laboratory and all technicians were blinded to patients’ disease progression status.

Plasma concentrations of LPS were determined with a commercial LAL kit (Kinetic-QCL; Bio Whittaker, Walkersville, MD, USA) for the quantitative determination of LPS using a known endotoxin standard as a reference (*E. coli* O55:B5 endotoxin). Plasma samples were diluted with endotoxin-free water and then heated to 90°C for 15 min to inactivate plasma proteins. The assay was carried out according to the protocol recommended by the manufacturer.

### Statistical analysis

The main characteristics of the study population of co-infected individuals have been described. Kaplan-Meier plots were used to investigate, the association of MT and IA with the following endpoints: i) time to single ALT >200 IU/l, ii) time to ≥2 consecutive observations of Fib-4 >1.45. Follow-up was censored at time of last clinical follow-up.. Moreover, all patients included in the analysis had a Fib-score <1.45 at baseline and none of them developed the liver disease or died of liver-related causes throughout the follow-up period.

An adjusted analysis was performed using a Cox regression model. Patients from whom more than one sample was obtained contributed to the analysis with more than one measurement; therefore, robust standard errors were used to control for non-independence of episodes coming from the same patient. Biomarkers were fitted in the models as categorical variables constructed using the median values observed in the co-infected as category-defining thresholds. Individuals, for whom no value could be attained from the sample, were included as a separate group. Several multivariable models, each including a single biomarker, were controlled for age, CD4, viral load (VL), baseline ALT, year of test, and duration of HIV infection at the date of the test. A further multivariable model including all biomarkers concomitantly was also fitted.

## Results

We analysed the data of 127 patients who had at least one measure of at least one of the evaluated biomarkers and contributing 373 measures in the survival analysis. 118 patients were co-infected with hepatitis C, and 9 with hepatitis B virus.

Table 1 shows the baseline characteristics of HIV-HBV/HCV co-infected patients. HIV-HBV/HCV co-infected patients displayed median HIV RNA load of 3.8 log10 copies/mL, median ALT levels of 40 IU/l. Sixty-nine percent of HIV/hepatitis co-infected patients were IDU and, at baseline, had been infected with HIV for a median of 5 years. Median activated CD8 + CD38 + HLA-DR + cells were 45% (IQR 25.5, 54.9), plasma TNF-α was 2.3 pg/mL (IQR 1.7, 3.4) and plasma IL-6 1.1 pg/mL (IQR 0.6, 2.1). Median circulating LPS and sCD14 levels were 126.2 pg/mL (IQR 75.0, 211.6) and 3.6 pg/mL, (IQR 2.0, 5.6), respectively.

**Table 1:** Baseline characteristic of HIV-HBV/HCV study population at the date of stored plasma

**Characteristics**	**N = 127**
Gender, n(%)	
**Female**	**45 (35.4%)**
Age, years	
**Median (range)**	**34 (22, 56)**
Mode of HIV transmission, n (%)	
**Homosexual contacts**	**17 (13.4%)**
**Heterosexual contacts**	**20 (15.7%)**
**IDU**	**87 (68.5%)**
**Other/unknown**	**3 (2.4%)**
Viral load, log10 copies/mL	
**Median (IQR)**	**3.8 (3.0, 4.3)**
CD4 count, cells/mmc	
**Median (range)**	**596 (208, 1303)**
Hepatitis co-infection, n (%)	
**HBV+/HCV-**	**9 (7.1%)**
**HCV+/HBV-**	**118 (92.9%)**
Time from HIV seroconversion, years	
**Median (range)**	**5 (0, 25)**
Calendar year of sample	
**Median (range)**	**1998 (1997, 2008)**
Biomarkers	
CD8CD38 + DR+, % (n = 120)	
**Median (IQR)**	**45.0 (25.5, 54.9)**
IL-6, pg/ml (n = 279)	
**Median (IQR)**	**1.1 (0.6, 2.1)**
LPS, pg/ml (n = 212)	
**Median (IQR)**	**126.2 (75.0, 211.6)**
sCD14, mg/ml (n = 294)	
**Median (IQR)**	**3.6 (2.0, 5.6)**
TNF-alfa, pg/ml (n = 286)	
**Median (IQR)**	**2.3 (1.7, 3.4)**
ALT, IU/l (n = 360)	
**Median (IQR)**	**40.0 (23.0, 63.0)**

**Figure 1:**
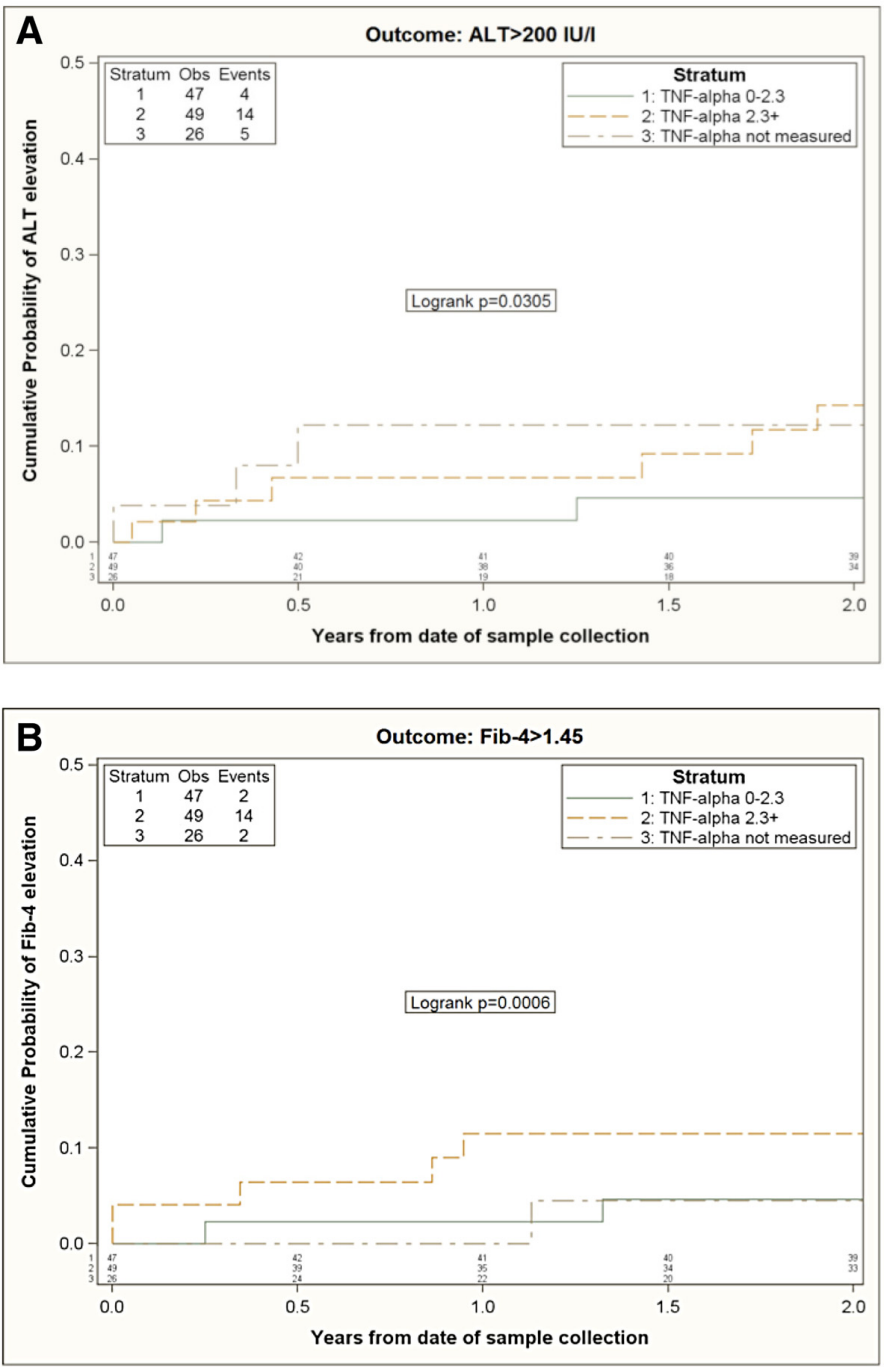
**Kaplan-Meier estimates of the risk of ALT elevation (A) and Fib-4 elevation (B) according to TNF-alpha strata.** Kaplan-Meier plots were used to compare the cumulative percent of patients reaching the primary endpoint. The cohort was stratified according to TNF-alpha plasma levels above or below the median (i.e. 2.3 pg/ml).

**Figure 2:**
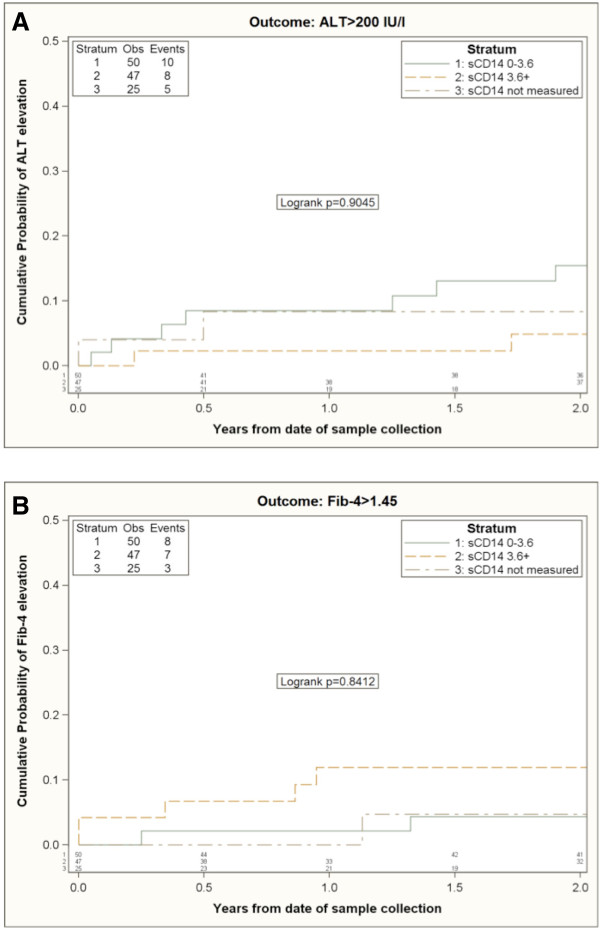
**Kaplan-Meier estimates of the risk of ALT elevation (A) and Fib-4 elevation (B) according to sCD14 strata.** Kaplan-Meier plots were used to compare the cumulative percent of patients reaching the primary endpoint. The cohort was stratified according to sCD14 plasma levels above or below the median (i.e. 3.6 pg/ml).

122/127 co-infected subjects were included in the time to ALT and time to Fib-4 elevation analysis according to TNF-a levels (Figure 1) and sCD14 levels (Figure 2). 5/127 co-infected patients for whom only baseline visit and no follow-up visit was available were excluded from survival analysis.

23/122 patients experienced ALT elevation and 18/122 had ≥ 2 consecutive observations of Fib-4 elevation above the selected threshold, respectively. Patients with higher circulating TNF-α were more likely to show a Fib-4 elevation. By 2 years from baseline, 12% (95% CI:2-22%) of individuals with a baseline TNF-α >2.3 pg/ml experienced the elevation vs. 5% (95% CI:0-11%) of those with TNF-α ≤2.3 pg/ml (log rank p = 0.0006).

When considering ALT > 200 IU/L as the measure of hepatitis disease progression, none of the biomarkers showed an independent association with the outcome, although subjects with TNF-α >2.3 pg/mL showed a 4-fold risk of progression in the unadjusted Cox regression model (vs. people with a value ≤2.3 pg/ml, p = 0.01); however, this was not confirmed in the adjusted model (Table 2).

**Table 2:** Relative hazards of ALT elevation >200 IU/l from fitting a Cox regression model

	**Crude and adjusted relative hazards of developing ALT>200 IU/l**					
**Biomarker**	**Crude RH (95% CI)**	**p-value**	Adjusted^* ^**RH (95% CI)**	**p-value**	Adjusted^** ^**RH (95% CI)**	**p-value**
**CD8CD38+DR+, %**						
<=48%	1.0		1.0		1.0	
>48%	0.30 (0.03, 2.93)	0.303	0.17 (0.01, 1.91)	0.150	0.09 (0.01, 1.44)	0.088
not measured	1.24 (0.37, 4.20)	0.726	1.12 (0.32, 3.94)	0.865	0.76 (0.18, 3.27)	0.711
**IL-6, pg/ml**						
<=1.1	1.0		1.0		1.0	
>1.1	2.77 (0.98, 7.87)	0.056	1.82 (0.61, 5.43)	0.281	1.56 (0.46, 5.32)	0.481
not measured	2.00 (0.61, 6.57)	0.251	1.79 (0.53, 6.07)	0.351	2.19 (0.18, 27.33)	0.543
**LPS, pg/ml**						
<=126	1.0		1.0		1.0	
>126	1.26 (0.44, 3.59)	0.669	0.77 (0.21, 2.83)	0.697	0.39 (0.08, 1.84)	0.235
not measured	0.66 (0.25, 1.78)	0.417	0.67 (0.22, 2.04)	0.478	0.31 (0.08, 1.28)	0.107
**sCD14, mg/ml**						
<=3.6	1.0		1.0		1.0	
>3.6	0.82 (0.32, 2.07)	0.669	0.79 (0.29, 2.19)	0.651	0.84 (0.25, 2.82)	0.778
not measured	0.98 (0.33, 2.86)	0.967	1.13 (0.35, 3.59)	0.842	0.80 (0.03, 25.38)	0.900
**TNF-alfa, pg/ml**						
<=2.3	1.0		1.0		1.0	
>2.3	4.01 (1.32, 12.21)	0.014	2.14 (0.64, 7.15)	0.217	2.03 (0.57, 7.29)	0.276
not measured	2.48 (0.67, 9.26)	0.175	1.82 (0.46, 7.17)	0.394	1.66 (0.10, 28.78)	0.728

^*^All models (a separate one for each biomarker) adjusted for age, CD4, VL, baseline ALT, year of sample, duration of HIV infection at the date of sample.

^**^Further mutually adjusted for all biomarkers.

In contrast, TNF-α levels higher than the median value were associated with a >8-fold higher risk of developing Fib-4 > 1.45 in both the unadjusted (p = 0.005), and adjusted analysis (p = 0.003) compared to people with a value below the median (Table 3).

**Table 3:** Relative hazards of Fib-4 elevation >1.45 from fitting a Cox regression model

	**Crude and adjusted relative hazards of developing Fib>1.45**					
**Biomarker**	**Crude RH (95% CI)**	**p-value**	Adjusted^* ^**RH (95% CI)**	**p-value**	Adjusted^** ^**RH (95% CI)**	**p-value**
**CD8CD38+DR+, %**						
<=48%	1.0		1.0		1.0	
>48%	5.27 (0.59, 47.23)	0.137	4.62 (0.47, 45.35)	0.189	5.34 (0.31, 92.47)	0.250
not measured	2.82 (0.37, 21.58)	0.318	2.88 (0.35, 23.80)	0.327	1.71 (0.19, 15.66)	0.636
**IL-6, pg/ml**						
<=1.1	1.0		1.0		1.0	
>1.1	1.67 (0.59, 4.68)	0.333	1.62 (0.52, 5.05)	0.403	1.24 (0.34, 4.58)	0.747
not measured	0.71 (0.18, 2.84)	0.627	0.56 (0.13, 2.46)	0.443	§	0.992
**LPS, pg/ml**						
<=126	1.0		1.0		1.0	
>126	0.41 (0.11, 1.60)	0.200	0.36 (0.07, 1.78)	0.211	0.53 (0.08, 3.33)	0.497
not measured	0.53 (0.19, 1.46)	0.217	0.64 (0.21, 1.96)	0.431	0.71 (0.13, 3.89)	0.697
**sCD14, mg/ml**						
<=3.6	1.0		1.0		1.0	
>3.6	0.99 (0.36, 2.74)	0.987	0.46 (0.14, 1.53)	0.206	0.20 (0.04, 0.90)	0.036
not measured	0.69 (0.18, 2.61)	0.582	0.33 (0.08, 1.46)	0.145	§	0.992
**TNF-alfa, pg/ml**						
<=2.3	1.0		1.0		1.0	
>2.3	8.37 (1.90, 36.87)	0.005	15.17 (2.72, 84.76)	0.002	13.05 (2.43, 70.07)	0.003
not measured	1.70 (0.24, 12.11)	0.596	1.53 (0.17, 13.71)	0.702	0.29 (0.01, 10.81)	0.503

^*^All models (a separate one for each biomarker) adjusted for age, CD4, VL, Fib, year of sample, duration of HIV infection at the date of sample.

^**^Further mutually adjusted for all biomarkers; § RH not obtained given small sample size for the parameter.

Most interestingly, in this same model, higher sCD14 levels were also independently associated with a 80% reduction in the risk of Fib > 1.45 (p = 0.04) (Table 3).

Indeed, in the univariable analysis, higher levels of sCD14 were associated with a slightly increased risk of Fib-4 elevation (Figure 2B). However, the estimated difference was small: 0.5% increase by 2 years comparing people with a sCD14 >3.6 mg/mL vs. ≤3.6 mg/ml using the Kaplan-Meier approach and a RH = 0.99 per (95%CI 0.36-2.74) using the Cox regression model (Table 3). However, after controlling for confounding factors the adjusted RH associated with a sCD14 >3.6 mg/mL was 0.20 (95%CI 0.04-0.9), p = 0.04. The major confounding factors seem to be the baseline Fib-4 value (higher in those with sCD14 >3.6 mg/mL, which is also a factor associated with greater risk of increasing Fib-4) LPS (inversely correlated with sCD14 and associated with lower risk of Fib-4 increase) and CD8CD38 + HLA-DR + values (positively correlated with sCD14 and associated with increased risk of Fib-4 increase). After controlling for these factors the RH was already modified to a value <1 (RH = 0.46, 95% CI: 0.14-1.52).

Similar results were obtained in a sensitivity analysis including only HIV/HCV co-infected patients (excluding HIV/HBV co-infected patients). One hundred and thirteen HIV/HCV co-infected individuals were included in this time to ALT and time to Fib-4 elevation sensitivity analysis. Interestingly, in the Kaplan-Meier analysis, patients with higher circulating TNF-a were more likely to display liver disease progression according to both endpoints, i.e. ALT > 200 IU/L (log rank p = 0.04) and Fib-4 > 1.45 (log rank p = 0.0004). Similar to the main analysis, compared to people with values below the median value, those with TNF-α levels higher than the median showed a >8-fold higher risk of developing Fib-4 > 1.45 in the adjusted analysis (RH 8.10, 95%CI 1.44, 45.68; p = 0.02); and sCD14 higher than the median was independently associated with a reduction in risk of Fib > 1.45 (RH 0.04, 95%CI 0, 0.67; p = 0.03).

## Discussion and conclusions

We sought out to investigate whether MT markers might be able to predict the progression of hepatic disease in a cohort of HIV-HBV/HCV co-infected patients. Because of the lack of liver-related clinical events in our study population we evaluated the risk of liver disease progression using the elevation of commonly employed surrogate markers.

In our cohort of HIV-HBV/HCV co-infected patients, higher TNF-α plasma levels were associated with increased risk of developing ALT > 200 IU/l (in the unadjusted analysis only) and Fib-4 > 1.45 (both in the unadjusted and adjusted analysis), supporting our *a priori* hypothesis that a pro-inflammatory status may induce disease progression in HIV/hepatitis co-infected patients, possibly as mediator of hepatic inflammation/angiogenesis.

Nevertheless, to our surprise, higher sCD14 levels were also independently associated with a decreased risk of Fib-4 elevation to a value >1.45. Our results appear to be in disagreement with those obtained in previous analyses showing a positive association between sCD14 and viral hepatic disease both in HIV-negative and HIV-positive individuals [5,9,12]; this may be partly explained by the different study design, patient populations and endpoints used to define liver disease progression. In particular, Redd *et al.* have recently described an association between elevated circulating sCD14 and liver stiffness. However, Redd *et al.* analysed a very different patient population of Ugandans HIV infected individuals with no viral hepatitis co-infections [13], who may feature different MT and systemic IA as compared to the HIV-viral hepatitis co-infected subjects included in our research.

Interestingly however, our findings are in accordance with other data reporting an association between lower circulating sCD14 and increased ALT levels in non-alcoholic fatty liver disease [16]. As a matter of fact, amongst its many functions, sCD14 also prevents the interaction of LPS with phagocyte membrane CD14, thus hampering the inflammatory response [17]. According to this alternative model, high sCD14 levels might preserve liver function by contributing to the down-regulation of the inflammatory cascade [16,18] which accounts for disease progression in HIV/hepatitis co-infected patients. As a possible counterpart to our data, it is intriguing to speculate that HIV/hepatitis co-infected subjects who are less likely to progress to liver disease (i.e. Fib-4 >1.45) maintain high circulating sCD14 that might exert a protective function against hepatic illness by neutralizing gut-derived LPS.

An alternative interpretation of our findings is that in this study liver damage was estimated by means of indirect measures (i.e. ALT or Fib-4 elevations) which are known not to be perfect surrogate markers for clinical disease. In particular, Fib-4 > 1.45 is accepted for F0-F1, but does not perform well in intermediate stages; however a Fib-4 value > 3.25 (a more reliable measure for intermediate fibrosis stages) was observed only in 3 patients, and, therefore, this more strigent definition of the endpoint could not be used in this analysis. Conversely, previous literature reports on HIV/HCV co-infected patients [5,9], including data from our group [12], described in detail the effects of MT in liver disease as assessed by histology and/or elastography measures. Given that hepatic stellate cells and Kupffer cells are the main target through which LPS promotes fibrogenesis [19], direct measures of liver disease may more faithfully mirror the effects of MT on hepatic tissues. The use of ALT/Fib-4 elevations might also explain the discrepancies between our results and the data by Redd *et al.* who also identified liver disease by means of transient elastography [13]. A further explanation to the difference between our findings and those by Redd’s is that our study design is prospective, and might be therefore less prone to selection bias than a case–control study.

The fact that the results of the analysis using ALT elevation as an endpoint are only in partial agreement with those found using Fib-4 elevation is obviously of slight concern. However with 23 and 18 events included in the analysis, respectively, it is difficult to discern between genuine differences and discrepancies due to chance circumstances or lack of power. This is a post-hoc analysis of a main protocol focusing on HIV-disease progression endpoint [7], not powered for hepatitis-disease endpoint and with no current plan to expand the study population by extracting additional samples from the repository. Nonetheless, from a more pathogenic standpoint, ALT levels are a measure of liver inflammation, and Fib-4 reflects hepatic fibrosis which does not necessarily occur at the same time. As a matter of fact, given that inflammation drives fibrosis, heightened ALT levels may occur prior to Fib-4 increases.

Several other limitations of our study have to be acknowledged. First of all, the design of our study is restricted to HIV-HBV/HCV co-infected patients and lacks a control group of HCV and/or HBV mono-infected individuals. Furthermore, the size of our cohort might not be large enough to allow to draw sure associations between MT and liver disease progression.

Nevertheless, given the significant clinical burden of hepatitis co-infection in HIV-positive patients, we believe that our findings provide a further insight in the understanding of the contribution of MT in HBV/HCV clinical outcome and may stimulate further research aimed to specifically investigate the mechanism(s) through which MT causes the progression of liver disease.

## Competing interests

The authors declare that they have no competing interests.

## Authors’ contributions

GM conceived the study, analysed data, wrote the paper; ACL conceived the study, performed all statistical analysis, substantially contributed to write the paper; CT participated to data analysis and helped in writing the paper; AC contributed to patients recruitment within the Icona Foundation Study group and edited the manuscript; FCS contributed to study design and patients recruitment within the Icona Foundation Study group and edited the manuscript; ADL contributed to study design and edited the manuscript; AA contributed to study design and patients recruitment within the Icona Foundation Study group and edited the manuscript; AC contributed to study design and patients recruitment within the Icona Foundation Study group and edited the manuscript; MP conceived the study, analysed data and substantially contributed to write and edit the paper; AdM conceived the study, analysed data and substantially contributed to write and edit the paper. All authors read and approved the final manuscript.

## Pre-publication history

The pre-publication history for this paper can be accessed here:

http://www.biomedcentral.com/1471-2334/14/79/prepub
